# Expensive lifesaving treatments: allocating resources and maximizing access

**DOI:** 10.1186/s13584-017-0195-7

**Published:** 2018-01-04

**Authors:** Rachel Nissanholtz-Gannot, David Chinitz

**Affiliations:** 1 Department of Health System Management, Ariel University, University Hill, 40700 Ariel, Israel; 2 Myers-JDC-Brookdale Institute, Jerusalem, Israel; 3 Department of Health Policy and Management, School of Public Health, Hebrew University-Hadassah, Jerusalem, Israel

## Abstract

Avisar et al. present an exemplary model for outreach aimed at ensuring that a maximum of patients eligible for expensive Hepatitis C (HPC) drugs receive treatment. We enlarge the picture to put their model in the political, economic and regulatory framework for financing and providing these drugs in Israel and a number of other countries. We then return to delivery system level and consider issues such as cost of outreach, the need for health care coordinators and dealing with Hepatitis C patients not yet entitled to receive the drugs under national health coverage determinations.

Regarding national coverage decisions, we find that countries such as Australia, New Zealand, the United Kingdom and Israel all extended coverage for Hepatitis C drugs, given the clear high effectiveness of the latter. However, to limit budget impact, all these countries target coverage to patients based on disease genotype and stage.

The model presented by Avisar et al., while impressive, leaves some items to address. These include: whether all resources allocated to HPC drugs are actually used for this purpose, the roles of outreach to HPC patients who do not meet the guidelines for treatment, and a comparison of the effectiveness of the model vs. a variety of costs associated with it.

The World Health Organization (WHO) estimated in July 2017 that there are 71 million people living with Hepatitis C (HPC) worldwide, with 399,000 dying from the disease annually [1]. HPC is a severe disease with high rates of mortality; however, during recent years its treatment has undergone dramatic change, as Direct Acting Antivirals (DAA) drugs^1^ have been found to significantly limit death from HPC.

Avisar et al. describe a systematic approach to ensuring access of HPC patients to DAA drugs in an Israeli health plan. In this commentary we view their article in the context of international policies regarding access to these drugs, and raise some regulatory and management issues that arise for health systems dealing with expensive, highly cost effective drugs targeted at specific, often vulnerable, populations.

DAA drugs enter the scene of priority setting in health care that has become a sub-field of its own since the mid 1990s. Health systems in developed countries have become more explicit in defining benefits baskets, in other words, what services are covered by universal health insurance programs. What used to be considered “tragic choices” [2] and “painful prescriptions” [3] that were well-nigh impossible to deal with in the open and in an accountable fashion, are now considered tough choices that can be made in a politically sustainable manner. There are a variety of national approaches to deciding what will be included in publicly financed baskets of health services [4], none of them perfect, but all seeming to cope with absorbing a never ending stream of new technologies within constrained budgets. With the help of technocratic evaluative approaches [5], together with visible political mechanisms for decision making [6, 7], politicians have, in some countries, bitten this large size bullet. The media and the public, while not always happy with the predicament or the decisions, is becoming accustomed and accepting of the need to set limits [6–8]. It is in this context that the appearance of a drug that is hugely expensive but also highly effective for a sizeable group of patients, is dealt with by national health systems.

At least three countries with universal health insurance coverage, namely, Australia, New Zealand and the United Kingdom have documented policies for HPC drugs. The health systems of these countries face the same challenge as the Israeli system. Comparison among them and to the Israeli case highlights the advantages and challenges facing universal health systems in dealing with access to HPC drugs.

Australia through its Pharmaceutical Benefits Scheme (PBS), the UK NHS through the National Institute for Clinical Excellence (NICE), and New Zealand through its Pharmaceutical Management Agency (Pharmac), have faced the issue of financing these expensive and highly effective drugs. In the case of Australia, with a quarter of a million HPC patients, access to DAA drugs was guaranteed for hepatitis genotypes 1 through 4, while patients with genotypes 5 and 6 are granted access to these drugs in combination with interferon and ribavirin [9].

In New Zealand, there are approximately 50,000 HPC cases, at a cost of between US $48,000 to $96,000 per treatment with DAA. Pharmac, based on cost effectiveness data, and negotiations with suppliers, made HPC drugs such as sofobuvir, ledipasvir/sfosbuvir and partiaprevir, combined with ritonavir and ombitasvir and dasbuvir available to patients meeting access criteria in 2016. [10]

In the UK, with 215,000 cases [11], NICE recommended three new treatment options for hepatitis C: daclatasvir, ledipasvir/sfosbuvir, and ombitasvir/paritaprevir/ritonavir with or without dasabuvir. According to the recommendations, these drugs should be administered according to patient genotype and liver disease stage. Patients receiving treatment outside of the guidelines, at the time of its publication, could continue the treatment until their treating physician considered it appropriate to stop. These recommendations were expanded in October 2016 and January 2017. The number of options of drugs choice were expanded and the guidelines also recommend that a multidisciplinary team will decide on the treatment. [12].

In Israel, DAA drugs are quite expensive (at least 130,000 NIS per patient) and ways have to be found to maximize benefit within limited budgets. The policy response has two steps. The first is to maximize financial coverage to HPC patients within budget constraints. The second is to ensure that HPC patients who have been given coverage actually receive the treatment.

The first step is accomplished through the mechanism of the Public Committee to Update the National Health Insurance (NHI) Basket of Services [6]. As described elsewhere, this committee allocates a pre-determined budgetary increment to new drugs and health technologies each year. Based on a combination of cost effectiveness criteria and public values, this allocation includes detailed indications for utilization of the services added to the basket. However, once a treatment is included for specified indications, patients meeting the relevant criteria are given free access to it, provided it has been recommended by qualified physicians. In the case of DAA drugs, the Ministry of Health (MOH) managed to reduce the price of treatment through negotiation with pharmaceutical companies, which increased the finance available for these drugs without this coming at the expense of other drugs and technologies that also are worthy of being included in the NHI basket of services.

These country examples highlight that the justifications for allocating resources to DAA drugs rest crucially on prioritizing HPC patients according to their clinical status. This brings into focus the second policy step; namely, ensuring that all patients whose clinical status qualifies them for treatment are in fact treated with DAA drugs. Carrying out the intended policies is the responsibility of the health care delivery system, and in particular, in Israel, the four Health Plans that cover the entirety of Israel’s population. Ever since the Black Report on the UK National Health Service in 1980 [13], it is well known that financial access does not guarantee actual utilization of health entitlements. Especially for vulnerable populations, wherein the incidence of HPC is disproportionately high, the system must engage in outreach in order to ensure appropriate uptake of services. When relatively large sums are allocated to DAA drugs, it is imperative to maximize their utilization, especially given their high levels of efficacy. Pharmac accompanied its decision to cover DAA drugs describe above with creation of an e-tool for clinicians to learn how to prescribe the medications [14].

The article by Avisar et al. presents an exemplary model for expediting access to HPC drugs in the context of an Israeli health plan. The Multi-disciplinary Patient Centered Model (MSPC) that it describes coalesces providers from different professions and organizational sub-systems, including managers, nurses, pharmacists, family physicians and specialists. This unique array, while developed in the context of one region, appears readily adaptable to other regions of the health plan, as well as to other Israeli health plans and delivery systems in other countries.

The very comprehensiveness of the model, however, highlights concerns not raised in the article. First, given the large sums of money involved, the question is posed whether it is necessary to monitor whether the resources allocated for DAA treatment are actually being used for that purpose. There are two options. One is to require financial reporting by health plans of exactly how much they are spending on these drugs. In the case of the Macabbi plan’s program as described, it is reasonable to assume that the outreach efforts lead to exhaustion of the relevant budgets. But if not, should policy makers hold plans accountable for this specific utilization of the funds, or assume that funds not used for DAA drugs are used for other services included in the NHI basket? While this conundrum applies to any drug or service in the basket, in the case of a dramatically effective treatment such as DAA for HPC, such monitoring may be considered particularly desirable.

Second, while the MSPC model strives to ensure access to DAA drugs for all patients meeting the relevant criteria, how does the health plan relate to HPC patients not so covered? Are steps necessary to explain to such patients that the delay in their access to the drugs will not unduly harm them? Overall, the Israeli public seems to accept the limitations of the process for expanding the NHI basket of services [6, 7]. But in the case of a highly effective and high profile treatment, learning about the perspective of untreated patients, and nurturing public understanding and maintaining public trust in coverage determinations, may be called for.

The HPC treatment model described in the article by Avisar et al. highlights the importance of better understanding the role of nurse coordinators, definition of the role, and development of policies for training and regulation of this evolving function, issues that have also been raised by others [15, 16].

Finally, and related to the previous point, the article by Avisar et al. does not discuss the costs of the MSPC model. A complete assessment of the cost effectiveness of HPC drugs should include the expensive costs of work force, organizational redesign, and evaluation linked to the MSPC model. It is perhaps likely that the coordinated, continuous and culturally appropriate aspects of the MSPC model will prove to be worth their cost, and also that the model will be relevant for application to other areas health service delivery to targeted groups. Still, the cost of organizational innovations such as MSPC needs to be accounted for.

## Conclusion

The MSPC model for expediting access to HPC drugs in Israel, while impressive, leaves some items to address. These include: whether all resources allocated to HPC drugs are actually used for this purpose, the roles of outreach to HPC patients who do not meet the guidelines for treatment, and a comparison of the effectiveness of the model vs. a variety of costs associated with it. Continuous evaluation of the model in the case of HPC in Macabbi, as well as in other settings and regarding other high cost/high effectiveness treatments, can shed light on these aspects and how they should be dealt with.

## Abbreviations

DAA: Direct acting antivirals drugs
HPC: Hepatitis C
MOH: Ministry of Health
MSPC: The Multi-disciplinary patient centered model
NHI: National health insurance law
NHS: National Health Service
NICE: National institute for clinical excellence
PBS: Pharmaceutical benefits scheme
Pharmac: Pharmaceutical management agency
UK: United Kingdom
WHO: The World Health Organization

## Footnotes

1 DAAs are molecules that target specific nonstructural proteins of the virus and this results in disruption of viral replication and infection. All the names of the different medications in the paper were taken from the references. The authors are not physicians, therefore, we do not refer to the mechanism of specifics drugs.
